# Effect of ivacaftor treatment in patients with cystic fibrosis and the G551D-CFTR mutation: patient-reported outcomes in the STRIVE randomized, controlled trial

**DOI:** 10.1186/s12955-015-0293-6

**Published:** 2015-07-02

**Authors:** Alexandra Quittner, Ellison Suthoff, Regina Rendas-Baum, Martha S. Bayliss, Isabelle Sermet-Gaudelus, Brenda Castiglione, Montserrat Vera-Llonch

**Affiliations:** Department of Psychology, University of Miami, 5665 Ponce de Leon Blvd, Coral Gables, FL 33146 USA; Vertex Pharmaceuticals Incorporated, 50 Northern Avenue, Boston, MA 02210 USA; Optum, 24 Albion Road, Bldg 400, Lincoln, RI 02854 USA; Hopital Necker Enfants Malades, Universite Paris Sorbonne, 149 Rue de Sèvres, Paris, 75015 France

**Keywords:** Cystic fibrosis, Ivacaftor, Patient-reported outcomes, CFQ-R, Health-related quality of life

## Abstract

**Background:**

Cystic fibrosis (CF) is an inherited, rare autosomal recessive disease that results in chronically debilitating morbidities and high premature mortality. We evaluated how ivacaftor treatment affected CF symptoms, functioning, and well-being, as measured by the Cystic Fibrosis Questionnaire-Revised (CFQ-R), a widely-used patient-reported outcome (PRO) measure.

**Methods:**

STRIVE, a double-blind, placebo-controlled randomized trial, evaluated ivacaftor (150 mg) in CF patients aged 12+ with the *G551D-CFTR* mutation for 48 weeks. Treatment effect analysis used a mixed-effects repeated measures model. Treatment benefit analyses applied the cumulative distribution function and a categorical analysis of change scores (“improvement,” “no change,” or “decline”). Content-based interpretation examined treatment effect on specific item responses.

**Results:**

Data from 152 patients with a baseline CFQ-R assessment were analyzed. The treatment effect analysis favored treatment with ivacaftor over placebo on the Body Image, Eating, Health Perceptions, Physical Functioning, Respiratory, Social Functioning, Treatment Burden, and Vitality scales. Findings were supported by the analysis of categorical change. On all CFQ-R scales, the percentage of patients who improved was greater for ivacaftor. In the content-based analysis, the treatment benefit was characterized by better scores across a broad range of domains.

**Conclusions:**

Results illustrate broad benefits of ivacaftor treatment across many domains: respiratory symptoms, physical and social functioning, health perceptions, and vitality, as measured by the CFQ-R. The breadth of improvements reflects the systemic mechanism of action of ivacaftor compared to other therapies. Findings support the patient-reported value of ivacaftor treatment in this patient population.

**Trial Registration:**

ClinicalTrials.gov NCT00909532

**Electronic supplementary material:**

The online version of this article (doi:10.1186/s12955-015-0293-6) contains supplementary material, which is available to authorized users.

## Background

Cystic Fibrosis (CF) is a rare, autosomal recessive disease affecting multiple organs, including the lungs, pancreas, sweat glands, and intestinal, biliary and reproductive tracts [1]. CF is caused by defects in the CF transmembrane conductance regulatory (CFTR) protein resulting from mutations in the *CFTR* gene. Some *CFTR* mutations produce *CFTR* protein channels that have defective gating (reduced channel open probability), resulting in little to no net chloride ion transport [2–4]. Patients with CF who have at least one *CFTR* mutation of this type are at high risk for early lung function decline and progression of other disease manifestations. The most prevalent *CFTR* mutation that predominantly affects *CFTR* channel open probability is *G551D*, which is found in approximately 4 % of patients with CF in the United States [5].

Ivacaftor (Kalydeco®) was approved by the FDA in January 2012 for the treatment of CF in patients 6 years of age and older who have the *G551D* mutation on at least one *CFTR* allele. The approval was expanded in the US in February 2014 and December 2014 to include 9 additional mutations [6]. All of the currently indicated mutations affect *CFTR* channel open probability as their primary molecular dysfunction.

The Phase 3 clinical program for ivacaftor was designed to assess the efficacy and safety of ivacaftor treatment in patients with at least one of the specified *CFTR* mutations. Treatment-related changes in patient-reported outcomes (PROs) were evaluated and have been previously reported for children (ENVISION study), and adolescents and adults (STRIVE study) [7, 8] with at least one *G551D-CFTR* mutation.

Developers of drugs are expected to provide direct evidence of the treatment benefit as experienced by patients. Providers, regulators, and payers seek information about how patients feel and function in daily life, with evidence from pivotal trials implementing well-defined and reliable assessments. To that end, we expand on data previously reported that show the impact of CF and its treatment on how patients feel and function in everyday life.

The objective of this study was to evaluate the treatment effects of ivacaftor on patient-reported symptoms and health-related quality of life (HRQoL) as reported by patients with CF 12 years of age and older with the *G551D*-*CFTR* mutation participating in the STRIVE study. The specific aim was to interpret treatment benefit using innovative analyses of PRO data covering a broad range of HRQoL domains and CF symptoms, specifically: 1) mean changes in Cystic Fibrosis Questionnaire-Revised (CFQ-R) scale scores from baseline through 48 weeks of treatment; 2) patterns of treatment response using empirically-defined categories of change; and 3) examination of treatment using responses to salient items of the CFQ-R that characterized disease impact.

## Methods

### Data source

STRIVE was a randomized, double-blind, placebo-controlled study evaluating ivacaftor, a *CFTR* potentiator, in subjects 12 years of age or older with CF and at least one *G551D-CFTR* mutation. Patients were randomly assigned in a 1:1 ratio to receive 150 mg of ivacaftor or placebo every 12 h for 48 weeks. Throughout the study, all patients continued to take their pre-study medications (with the exception of hypertonic saline). The primary endpoint was the estimated mean absolute change from baseline through week 24 in the percent predicted forced expiratory volume in 1 s (FEV_1_ % predicted). Secondary endpoints included the change from baseline through week 48 in the FEV_1_ % predicted, time to first pulmonary exacerbation, change in body weight, change in concentration of sweat chloride, as well as patient-reported scores from the CFQ-R Respiratory Symptoms Scale [9–11].

The study included a 2-week screening period, a 2-week run-in period, and a 48-week treatment period. Efficacy and safety findings from this study have been previously reported [9].

### Cystic fibrosis questionnaire-revised

The CFQ-R was the first disease-specific PRO instrument developed for use by patients with CF and their caregivers [12]*.* The original CFQ was developed from qualitative and quantitative studies which included a conceptual framework, interviews with patients, parents, and health care professionals, cognitive testing, and psychometric evaluation [10, 12–14]. Modifications were subsequently made to the instrument leading to the current revised version, which has been identified as a widely used PRO measure for CF [15–17].

Three versions of the CFQ-R are available: a self-completed *Teen/Adult* version for patients with CF aged 14 and older, a *Child* version for children aged 6–13 years (self-report for ages 12–13 and interviewer-administered for ages 6–11), and a proxy-completed *Parent/Caregiver* version. The Teen/Adult and Parent/Caregiver versions include the following scales: Body Image, Digestive Symptoms, Eating Problems, Emotional Functioning, Health Perceptions, Physical Functioning, Respiratory Symptoms, Role Functioning, Social Functioning, Treatment Burden, Vitality, and Weight. The Child version does not include Health Perceptions, Role Functioning, Vitality, and Weight. Items included in each scale of the CFQ-R are summed and standardized to a 0–100 scale, with higher scores indicating better outcomes or fewer symptoms from the patient perspective.

In the STRIVE study, the CFQ-R was administered at the beginning of the run-in period, baseline, day 15, week 8, and every 8 weeks thereafter, up to week 48. Patients aged 14 and older completed the Teen/Adult version while those aged 12 and 13 at baseline completed the Child version. Parents of children aged 12 and 13 at baseline also completed the Parent/Caregiver version. This analysis included pooled data from the two self-completed Teen/Adult and Child versions (data from the Parent/Caregiver version were not included).

### Analyses

#### Treatment-related changes in CFQ-R domains

For each scale of the CFQ-R, mean changes from baseline through week 48 were evaluated by treatment group using a mixed-effects model for repeated measures [18] with absolute change from baseline as the dependent variable, fixed effects for study visit and treatment group, and adjustment for continuous baseline values of age, FEV_1_ % predicted, and baseline scale score, using an unstructured covariance matrix. Treatment effect was calculated as the difference in mean change from baseline between the ivacaftor and placebo groups. A *p*-value of <0.05 was employed to assess statistical significance.

In addition to evaluating treatment benefit as mean change from baseline, the current analyses also included evaluating treatment group differences in terms of the percentage of patients reporting various levels of change across the different CFQ-R domains. First, to evaluate treatment group differences across the entire range of observed change, we used a method based on the cumulative distribution function (CDF). The CDF of change scores presents the proportion of patients who experienced an improvement or decrement at or below a specific value [19]. When presented separately by treatment group, CDF plots illustrate the separation between groups at each threshold of change. For evaluating treatment response, CDFs are most easily interpreted when the y-axis is reversed to show the proportion of patients with a score change > |X|*,* an approach known as cumulative response curves (CRCs) [20]. We evaluated the CRCs for change from baseline to week 48 by CFQ-R scale, by treatment group. Statistical significance was assessed using the two-sample Kolmogorov-Smirnov test.

In addition to the CRC analysis, we used a distribution-based methodology [21] to establish threshold values that help interpret the magnitude of change observed in HRQoL scores. One-half standard deviation (SD) of the change from beginning to end of the 2-week run-in period (2 weeks prior to study drug initiation) was used as a threshold value [22] to identify minimal important change. The direction and magnitude of score changes were used to analyze treatment response. Positive or negative changes from baseline to week 48 exceeding (in absolute value) the threshold were considered “improvements” or “declines”, respectively. Change scores smaller (in absolute value) than the threshold value were considered “no change”. The Chi-square test was used to assess statistical significance of treatment group differences.

Sensitivity analyses were conducted using the standard error of measurement (SEM). The SEM reflects the measurement precision and the amount of random variation from repeated assessments. One SEM has been proposed as a measure of minimal clinically important difference (MCID) and was used to identify “improvement”, “no change”, or “decline” [23], with the Chi-square test for assessment of group differences. The SEM was evaluated as $SEM=S{D}_{\varDelta}\ast \sqrt{\left(1-ICC\right)}$, where *SD*_∆_ is the SD of change scores from the run-in period and the ICC is the intraclass correlation coefficient, a measure of reliability related to the repeated administration of the same test under the assumption that substantial change in the concept of interest has not occurred.

#### Content-based interpretation of treatment effects

These analyses examined the impact of treatment on responses to specific “sentinel” CFQ-R items empirically selected to represent each scale. Polyserial or Spearman [24] correlations between change in each item and change in its scale were calculated. The item with the strongest association with its scale change score was identified as the sentinel item. Response options for each sentinel item were collapsed into “none” vs. “any” impairment. The change in percent of patients with no impairment was evaluated by treatment group at baseline and week 48.

### Ethics

The STRIVE clinical trial (“A Phase 3, Randomized, Double-Blind, Placebo-Controlled, Parallel-Group Study to Evaluate the Efficacy and Safety of VX-770 in Subjects with Cystic Fibrosis and the G551D Mutation”) protocol was reviewed and approved by the institutional review board at each participating center, and each subject provided written informed consent or written or oral assent.

## Results and discussion

### Characteristics of study sample

The STRIVE study randomized 167 subjects to two treatment arms. Of these, 161 subjects subsequently received at least one dose (intention-to-treat analysis). This study sample contained the 152 patients who completed a baseline CFQ-R assessment. The two treatment groups were similar in terms of gender (53.0 % female) and mean age (25.7 years). Most patients were 18 years of age or older (77.6 %). At baseline, the treatment groups also were similar in average FEV_1_ % predicted (64.6 %), mean sweat chloride (100.4 mmol/L), height (167 cm), body weight (78.9 kg), and body mass index (BMI) (21.9 kg/m^2^).

### Treatment-related changes in CFQ-R scales

**Fig. 1:**
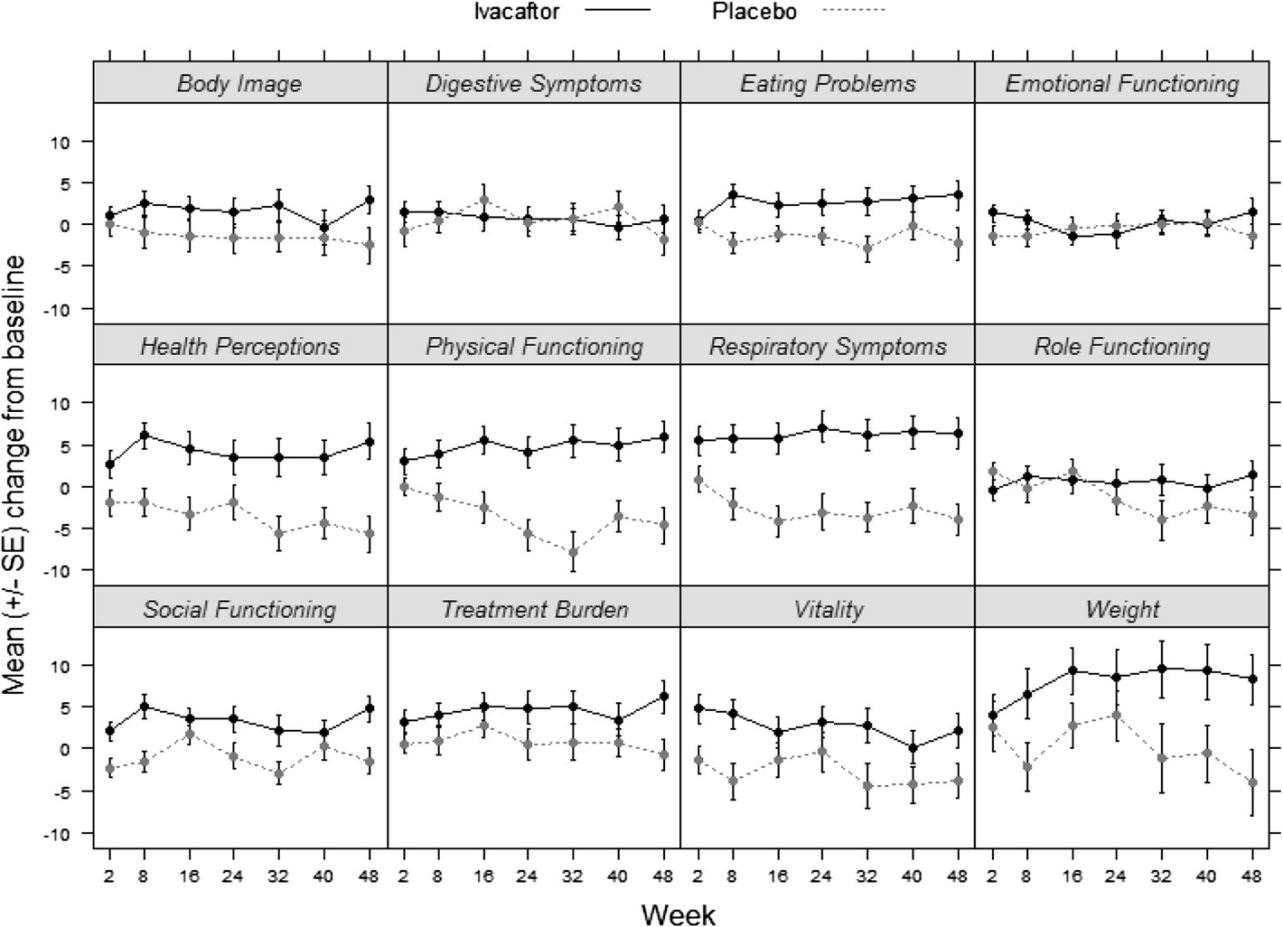
Change from baseline in CFQ-R scores for each visit by treatment group (observed data). SE = standard error; means and standard error are unadjusted

Figure 1 shows the observed mean and standard error of the change from baseline at each study visit, by scale and treatment group. Mean change on the Body Image, Eating Problems, Health Perceptions, Physical Functioning, Respiratory Symptoms, Social Functioning, Treatment Burden, and Vitality scales was consistently higher for ivacaftor vs. placebo. Favorable effects of ivacaftor were observed within the first 2 months of treatment initiation.

Table 1 presents baseline score, mean change from baseline through week 48, and treatment effects (difference between treatment groups in mean change) by CFQ-R scale and treatment group. For seven of the twelve CFQ-R scales, the mean post-baseline change was statistically significant, after adjustment for age, FEV1 % predicted, and baseline CFQ-R scale score. The largest treatment effect was found on the Respiratory Symptoms scale with a mean improvement of 5.9 points observed among patients receiving ivacaftor, exceeding the MCID value of 4 points, vs. a mean decline of 2.7 points among those receiving placebo (treatment effect of 8.6 points, *P* < 0.001) [12].

**Table 1:** CFQ-R adjusted change scores from baseline through week 48 by treatment group

CFQ-R Scale	Placebo		Ivacaftor			
	(*N* = 70^b^)		(*N* = 80^b^)			
	Baseline	Change	Baseline	Change	Treatment effect	*p*-value^a^
Body Image	80.3	−1.2	81.0	1.5	2.7	0.086
Digestive Symptoms	85.4	0.4	85.2	0.8	0.5	0.732
Eating Problems	91.9	−1.1	91.8	2.2	3.3	0.002
Emotional Functioning	83.6	−1.4	86.0	0.7	2.1	0.096
Health Perceptions	71.7	−3.6	72.1	4.0	7.6	<0.001
Physical Functioning	80.2	−1.7	76.1	2.7	4.4	0.006
Respiratory Symptoms	68.5^c^	−2.7	70.2	5.9	8.6	<0.001
Role Functioning	85.9	0.1	86.3	−0.5	−0.6	0.651
Social Functioning	71.9	−1.0	72.1	3.3	4.3	0.003
Treatment Burden	65.7	1.0	64.5	4.3	3.3	0.042
Vitality	64.7	−2.8	64.3	2.7	5.5	0.002
Weight	78.1	1.7	79.0	6.9	5.3	0.053

Health Perceptions, Role Functioning, Vitality and Weight are not included in the Child self-report version of the CFQ-R; results for these scales are based on the Teen/Adult version only (*N* = 64 for placebo and *N* = 76 for Ivacaftor)

^a^ *P*-value for overall post-baseline treatment effect, estimated using a mixed-effect model for repeated measures with fixed effects for study visit, treatment group, and adjustment for continuous baseline values of age, percent predicted FEV_1_ and domain score

^b^Analysis sample includes patients with a baseline assessment and at least one post-baseline assessment

^c^ *n* = 71

**Fig. 2:**
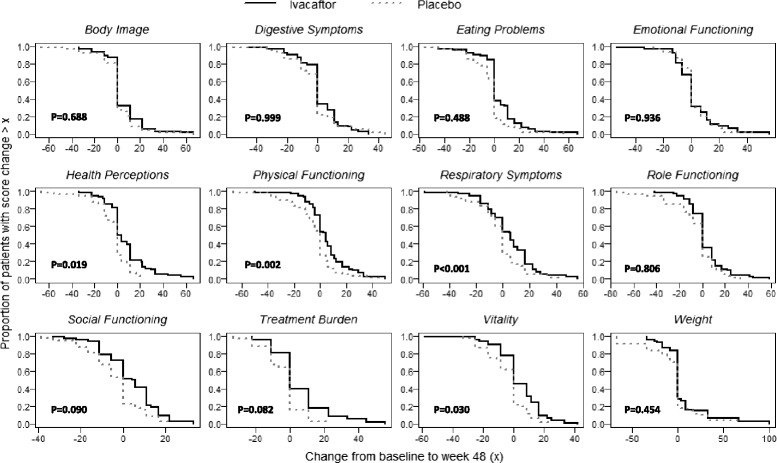
Cumulative response curves for CFQ-R scales by treatment group. *P*-values from the Kolmogorov-Smirnov two-sample test for equality of cumulative distribution functions

Figure 2 presents CRCs of change scores from baseline to week 48, by treatment group. For nearly all CFQ-R scales, lower cumulative change scores (worse outcomes) were seen for the placebo group, indicated by the dashed curve for the placebo group appearing to the left of the solid curve for ivacaftor. These findings suggest that the proportion of subjects who exceeded a particular level of change (better outcomes) was higher in the ivacaftor than placebo group. Differences between treatment groups (favoring ivacaftor) were greatest for Respiratory Symptoms (*p* < 0.001), Physical Functioning (*p* = 0.002), Health Perceptions (*p* = 0.019), and Vitality (*p* = 0.030). Using illustrative values of 5 and 10 points, the percentage of patients with change scores greater than 5 on the Respiratory Symptoms scale was 59 % for ivacaftor vs. 27 % in the placebo group. For changes greater than 10 points, percentages were 47 and 11 % for ivacaftor and placebo, respectively. For change scores of 5 and 10 points on Physical Functioning, percentages of ivacaftor-treated patients with improvement were 36 and 23 %, respectively, compared to 13 and 8 % in the placebo group.

**Fig. 3:**
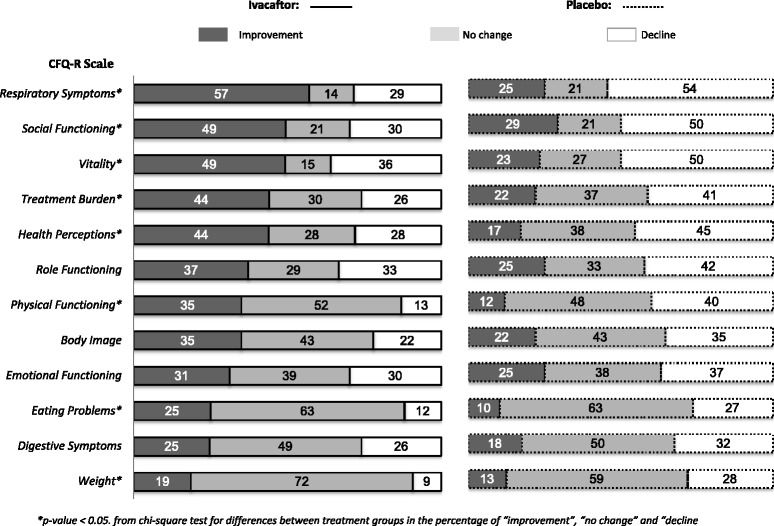
Analysis of categorical change from baseline to week 48 by CFQ-R scale and treatment group. *p-value < 0.05. from chi-square test for differences between treatment groups in the percentage of “improvement”, “no change” and “decline” patients

Figure 3 presents the percentage of patients who experienced “improvement”, “no change”, or “decline” after 48 weeks of treatment with either ivacaftor or placebo, according to our change threshold of 0.5 SD. Differences favoring ivacaftor were observed for Respiratory Symptoms (*P* < 0.001), Social Functioning (*P* = 0.026), Vitality (*P* = 0.006), Treatment Burden (*P* = 0.016), Health Perceptions (*P* = 0.003), Physical Functioning (*P* < 0.001), Eating Problems (*P* = 0.015), and Weight (*P* = 0.015).

Results were similar under the 1 SEM criterion (see Additional file 1: Tables S1 and Additional file 2: Table S2).

### Content-based interpretation of treatment effects

Table 2 shows the sentinel items by CFQ-R scale and the baseline percentage of patients reporting “no impairment”.

**Table 2:** Percentage of patients with “no impairment” in representative CFQ-R items at baseline and 48 weeks

	Representative items			% No Impairment at baseline (pooled)
CFQ-R scale	Teen-adult version/Child version	Response option^a^	Corr.^b^ with domain change score	
Body image	Look different from others	Very false/Not at all true	0.82	60.0
Digestive symptoms	Problems with gas/stomach hurt	Never	0.85^c^	48.1
Eating problems	Force myself to eat/pushed to eat	Very false/Never	0.82^c^	77.7
Emotional functioning	Felt sad/worried	Never	0.79	71.1
Health perceptions	Feel healthy	Very true	0.86	32.5
Physical functioning	Walking as fast as others	No difficulty	0.85	65.9
Respiratory symptoms	Coughing	Not at all/Never	0.85	3.7
Role functioning	Running errands out of the house	Never	0.85	41.0
Social functioning	Comfortable going out/got together with friends	Very true	0.66	59.2
Treatment burden	Tx makes life more difficult/Tx bothered you	Not at all/Not at all true	0.78	25.1
Vitality	Felt exhausted/grouchy	Never	0.81	47.4
Weight^d^	Trouble gaining weight	Not at all	^e^	62.0

^a^Identified to represent patient report of no impairment

^b^Polyserial correlation unless otherwise noted

^c^Spearman correlation

^d^Single item scale

^e^Not applicable for single item scales

Pooled = ivacaftor and placebo

The proportion of patients treated with ivacaftor who showed “no impairment” generally *increased* between baseline and week 48 and *decreased* among patients treated with placebo (data not shown). The items with at least 10 % separation between ivacaftor and placebo at week 48 were: *“feel healthy”* (17.5 % separation), *“walking as fast as others”* (15.1 %), *“coughing”* (14.1 %), *“going out with friends”* (14.1 %), *“trouble gaining weight”* (14.0 %), *“force myself to eat”* (13.5 %), and *“treatment makes life more difficult”* (12.5 %).

**Fig. 4:**
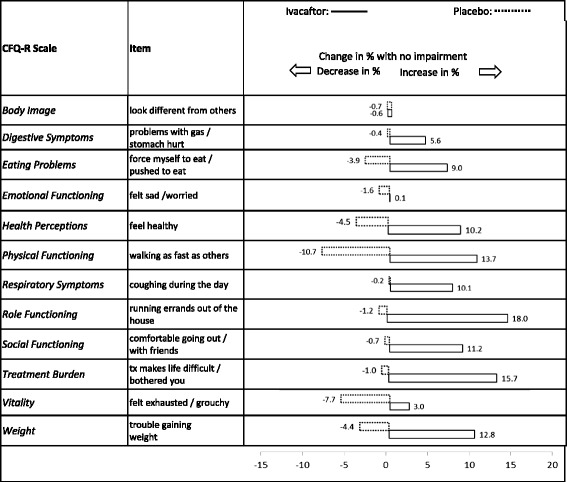
Change in the percentage of patients with no impairment after 48 weeks of treatment

Figure 4 presents the change in percentage with “no impairment” from baseline to week 48 by treatment group. As before, the percentage with “no impairment” increased with ivacaftor and decreased with placebo between baseline and week 48 for most items, eg, *“walking as fast as others”(+*13.7 vs. −10.7 %, respectively), *“feel healthy”* (+10.2 vs. −4.5 %), *“running errands out of the house”* (+18.0 vs. −1.2 %), *“going out with friends”* (+11.2 vs. −0.7 %), *[never feeling] “bothered by treatment”* (+15.7 vs. −1.0 %).

## Conclusions

Our analyses provide empirical data describing and quantifying the benefits of ivacaftor treatment in patients aged 12 years and older with CF and the *G551D*-*CFTR* mutation.

In the STRIVE study, early and sustained treatment effects from ivacaftor were observed through week 48 on the CFQ-R Respiratory Symptom scale. The magnitude and significance of the improvements in Respiratory Symptoms was consistently observed across the analyses in line with improvements in % predicted FEV_1_ in the study population. Of interest, sustained improvements were also observed on other scales of the CFQ-R, including Physical Functioning, Social Functioning, Eating Problems, Treatment Burden, Health Perceptions, and Vitality. Trends also were favorable for ivacaftor-treated patients on the Body Image, Digestive Symptoms, Role Functioning, and Emotional Functioning scales, but their magnitude was smaller and failed to reach statistical significance.

These positive changes across multiple CFQ-R scales are supported by findings from categorical change analyses. Using change scores classified into “improvement”, “no change”, or “decline”, the treatment effects favoring ivacaftor over placebo became even more apparent. On all 12 CFQ-R scales, the percentage who improved by week 48 was greater for ivacaftor-treated patients. Further, the percentage who declined was greater for placebo-treated patients, although differences across the three categories of change were not statistically significant for Role Functioning, Body Image, Emotional Functioning, and Digestive Symptoms. For the Respiratory Symptoms and Physical Functioning scales, the ivacaftor group included at least five times more patients with improvements compared to placebo. For the Social Functioning, Vitality, Treatment Burden, and Role Functioning scales, the ivacaftor group included three to five times more patients with improvements compared to placebo. Worsening scores through week 48 were more prevalent among patients on placebo. Of particular interest are the Weight scores, with a three-fold difference between ivacaftor and placebo in the percentage of patients who declined during the study (9 vs. 28 %).

The content-based analysis of sentinel items-showed that ivacaftor led to improvements in a broad range of functional outcomes, including the respiratory, nutritional, physical, social, and treatment-related domains.

Although several trial-based publications have documented treatment benefits using the CFQ-R, they have focused on selected scales: Respiratory Symptoms, Physical Functioning, and Vitality [25–30]. Additional data, particularly from observational studies, would provide needed real-world evidence to complement trial-based data on the patient-reported benefits associated with ivacaftor treatment. In the PERSIST open-label extension study sample, including 75 % (*n* = 144) of the STRIVE sample, the effect of ivacaftor on the CFQ-R Respiratory Symptoms scale was maintained over an additional 96 weeks of treatment [31]. In an observational study of ivacaftor (US GOAL), clinically meaningful and statistically significant gains (7.4 points, *p* < 0.0001) were observed in the Respiratory Symptoms scale after 6 months of treatment [32].

Clinicians, patients, and other stakeholders will benefit from understanding the impact of ivacaftor treatment on patients with selected CF mutations using a patient-centered point-of-view. We have presented direct evidence of therapy effects, extending the interpretation of treatment benefit beyond the previously reported clinical markers. These results complement the clinical results, providing evidence of how patients feel and function in daily life. Our findings suggest that ivacaftor treatment led to significant improvements that were substantial, sustained over 48 weeks, and spanned a wide range of symptoms, functioning, and well-being in patients with the *G551D-CFTR* mutation in the STRIVE study.

Results from the STRIVE study illustrate broad benefits of ivacaftor treatment across highly salient aspects of HRQoL: respiratory symptoms, physical and social functioning, health perceptions, and vitality as measured by the CFQ-R in patients with CF 12 years of age and older with the *G551D-CFTR* mutation. The breadth of improvements reflects the systemic mechanism of action of ivacaftor compared to other (symptomatic) therapies. Our results are supportive of the patient-reported value of ivacaftor treatment in this patient population.

Limitations: The analyses reported here are *post-hoc* in nature (not pre-specified). Although an MCID has been established for the Respiratory Symptoms scale [12], no such benchmarks for interpretation of change exist for the remaining CFQ-R scales. Further, we used distribution-based methods to identify thresholds of change [21] because use of an anchor-based method was not possible using these data. The STRIVE study included only patients with the *G551D-CFTR* mutation. As noted, our findings are based on results of a clinical trial and the magnitude of treatment effects of ivacaftor on patient-reported outcomes may differ in actual clinical practice.

## Additional files

Additional file 1:
**Categorical change from baseline-week 48 by CFQ-R scale and treatment group: 0.5 SD threshold.**
Additional file 2:
**Categorical change from baseline-week 48 by CFQ-R scale and treatment group: SEM threshold.**
