# Supplementary material for: Effect of ivacaftor treatment in patients with cystic fibrosis and the G551D-CFTR mutation: patient-reported outcomes in the STRIVE randomized, controlled trial
Source: Health Qual Life Outcomes. 2015 Jul 2;13:93. doi: 10.1186/s12955-015-0293-6 (PMC4702321; doi:10.1186/s12955-015-0293-6)
Supplement: Additional file 1: — Categorical change from baseline-week 48 by CFQ-R scale and treatment group: 0.5 SD threshold. [file 12955_2015_293_MOESM1_ESM.docx]

Additional file 1: Table S1. Categorical change from baseline-week 48 by CFQ-R scale and treatment group: 0.5 SD threshold

| CFQ-R scale | 0.5 SD* | Ivacaftor | | | | Placebo | | | | p-value^(b)^ |
| --- | --- | --- | --- | --- | --- | --- | --- | --- | --- | --- |
|  |  | **Improvement** | **No Change** | **Decline** |  | **Improvement** | **No Change** | **Decline** |  |  |
|  |  | **%** | **%** | **%** | **N^(a)^** | **%** | **%** | **%** | **N^(a)^** |  |
| *Body Image* | 6.5 | 35.1 | 42.9 | 22.0 | 77 | 22.1 | 42.6 | 35.3 | 68 | 0.114 |
| *Digestive Symptoms* | 5.1 | 24.7 | 49.3 | 26.0 | 77 | 17.6 | 50.0 | 32.4 | 68 | 0.511 |
| *Eating Problems* | 4.1 | 24.7 | 63.6 | 11.7 | 77 | 10.3 | 63.2 | 26.5 | 68 | 0.015 |
| *Emotional Functioning* | 4.9 | 31.1 | 39.0 | 29.9 | 77 | 25.0 | 38.2 | 36.8 | 68 | 0.604 |
| *Health Perceptions* | 6.5 | 44.0 | 28.0 | 28.0 | 75 | 17.2 | 37.5 | 45.3 | 64 | 0.003 |
| *Physical Functioning* | 4.5 | 35.1 | 51.9 | 13.0 | 77 | 11.8 | 48.5 | 39.7 | 68 | <0.001 |
| *Respiratory Symptoms* | 5.3 | 57.1 | 14.3 | 28.7 | 77 | 25.0 | 20.6 | 54.4 | 68 | <0.001 |
| *Role Functioning* | 5.4 | 37.3 | 29.3 | 33.3 | 75 | 25.0 | 32.8 | 42.2 | 64 | 0.284 |
| *Social Functioning* | 4.8 | 49.3 | 20.8 | 29.9 | 77 | 29.4 | 20.6 | 50.0 | 68 | 0.026 |
| *Treatment Burden* | 6.6 | 44.1 | 29.9 | 26.0 | 77 | 22.1 | 36.8 | 41.1 | 68 | 0.016 |
| *Vitality* | 5.7 | 49.3 | 14.7 | 36.0 | 75 | 23.4 | 26.6 | 50.0 | 64 | 0.006 |
| *Weight* | 12.1 | 18.7 | 72.0 | 9.3 | 75 | 12.5 | 59.4 | 28.1 | 64 | 0.015 |

*SD = standard deviation of change score during the run-in period (day 14 to baseline)

^(a)^ Patients with a baseline assessment and an assessment at week 48

^(b)^ p-value from chi-squared test for differences between treatment groups in the percentage of “improvement”, “no change” and “decline” patients
